# Stochastic Frontier Model Approach for Measuring Stock Market Efficiency with Different Distributions

**DOI:** 10.1371/journal.pone.0037047

**Published:** 2012-05-17

**Authors:** Md. Zobaer Hasan, Anton Abdulbasah Kamil, Adli Mustafa, Md. Azizul Baten

**Affiliations:** 1 Mathematics Section, School of Distance Education, Universiti Sains Malaysia, Penang, Malaysia; 2 School of Mathematical Sciences, Universiti Sains Malaysia, Penang, Malaysia; 3 Department of Decision Science, School of Quantitative Sciences, Universiti Utara Malaysia, Sintok, Darul Aman, Malaysia; Cinvestav-Merida, Mexico

## Abstract

The stock market is considered essential for economic growth and expected to contribute to improved productivity. An efficient pricing mechanism of the stock market can be a driving force for channeling savings into profitable investments and thus facilitating optimal allocation of capital. This study investigated the technical efficiency of selected groups of companies of Bangladesh Stock Market that is the Dhaka Stock Exchange (DSE) market, using the stochastic frontier production function approach. For this, the authors considered the Cobb-Douglas Stochastic frontier in which the technical inefficiency effects are defined by a model with two distributional assumptions. Truncated normal and half-normal distributions were used in the model and both time-variant and time-invariant inefficiency effects were estimated. The results reveal that technical efficiency decreased gradually over the reference period and that truncated normal distribution is preferable to half-normal distribution for technical inefficiency effects. The value of technical efficiency was high for the investment group and low for the bank group, as compared with other groups in the DSE market for both distributions in time- varying environment whereas it was high for the investment group but low for the ceramic group as compared with other groups in the DSE market for both distributions in time-invariant situation.

## Introduction

For investigating technical efficiency of financial institutions, most researchers used either parametric stochastic frontier approach (SFA) or non-parametric data envelopment analysis (DEA) [1]. Many researchers made a comparative study of parametric and non-parametric techniques for assessing the efficiency of financial institutions, for example banking industry [2]–[8] and insurance industry [9], [10]. From empirical evidence it is seen that DEA and SFA, which differ both in structure and implementation, provide significantly different efficiency scores. SFA employs a composed error model in which inefficiencies are assumed to follow an asymmetric distribution, usually the half-normal, while random errors are assumed to follow a symmetric distribution, usually the standard normal [11].

Most past studies used the half-normal and truncated normal distributions as assumptions about inefficiency effects model because of the ease of estimation and interpretation [12]. Truncated-normal distribution for inefficiency may be more appropriate than half-normal distribution [13]. Application of different distributions, like gamma and exponential, can be significant sometimes to the average efficiencies available for financial institutions [14]–[16]. This empirical study of SFA also used both half-normal and truncated normal assumptions on the inefficiencies and random error, as these are the most common assumptions in literature.

Dhaka Stock Exchange (DSE), by virtue of being the main stock exchange of Bangladesh, has significant implications for the performance of financial sector, and even the economy as a whole [17]. The focus of this study has been on the DSE, because it is not only the country's oldest stock exchange, but also one of the frontier emerging markets of South Asia according to Standard and Poor's Emerging Stock Markets Fact Book 2000. Studies concerning the market efficiency of DSE are available in [18], [19]. In particular, the linear relationship between share price and interest rate on DSE was studied by [20], through ordinary least square (OLS) regression.

In this study, for measuring the technical efficiencies of selected companies of DSE market of Bangladesh, SFA was used instead of DEA, because it has the advantage of dealing with stochastic noise, allowing for statistical tests of hypotheses concerning production structure and degree of inefficiency. Further, DEA does not impose any assumptions about production functional form and also does not take into account random errors; hence, the efficiency estimates may be biased if the production process is largely characterized by stochastic elements [21]. This study considered the stochastic frontier model for technical inefficiency effects in stochastic frontier production function proposed by [22]. This model was preferred, because in this study no explanatory variables were associated with technical inefficiency effects. Further, because this model was proposed for the analysis of panel data, Translogarithmic and Cobb-Douglas production frontier were used in empirical studies on production, including frontier analysis.

Finally, a time-varying inefficiency effects measure was used assuming truncated normal and half normal distributions [22]. The goal of this study is to identify the determinants that influence the share prices in DSE and the level of influence. Besides, it seeks to find out if factors, such as market return, market capitalization, book-to-market ratio and market value are significantly related to stock returns. This study is important, because it examines not only the capital market behavior of Bangladesh over the period 2000–2008 but also predicts the technical efficiencies for the selected groups of companies. At the same time, it is desirable to check whether technical efficiency is time variant or time invariant. Thus, this study is expected to provide meaningful insights into company's group-specific technical efficiency.

### Background of Dhaka Stock Exchange (DSE) Market

The Dhaka Stock Exchange market was established as “East Pakistan Stock Exchange Association Limited” on 28th April, 1954, but formal trading of the market began only in 1956. The name of the stock exchange was changed to “Dacca Stock Exchange Ltd” on 13th May, 1964, and service on the stock exchange continued uninterruptedly until 1971. The trading was suspended during the liberation war and resumed in 1976 with revised economic policy of the government. The Securities and Exchange Commission (SEC), which is the regulator of the capital market of Bangladesh, was established on 8th June, 1993 under the Securities and Exchange Commission Act, 1993. Later, on August 21, 2005, the DSE upgraded its automated trading system.

There are four types of market in DSE: (1) Public Market (2) Spot Market (3) Odd lot Market and (4) Block Market. The number of securities listed in the DSE market, including debentures and bonds, was 444 as on 25th November, 2010. The DSE has three indices: (1) DSI Index (comprises all listed securities of the exchange, calculated since November 01, 1993) (2) DSE General Index (comprises all companies, excluding the Z-category companies, started on November 27, 2001) and (3) DSE20 Index (comprises leading 20 shares with a base index of 1000 that was introduced on January 01, 2001) (Sources: DSE website: www.dsebd.org; and SEC website: www.secbd.org).

However, Dhaka Stock Exchange has been relentlessly trying to make the securities market an efficient reliable transparent organization that meets the challenges of economic reality of the country and makes the capital market as the center for economic development of the nation (Report on Dhaka Stock Exchange School of Business, University of Information Technology and Sciences, Dhaka, Bangladesh).

## Materials and Methods

### Stochastic Frontier Model with Technical Efficiency Effects

The stochastic frontier model for panel data can be written thus:
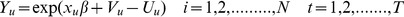
(1)where 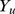 denotes the output for the i-th company in the t-th time period, 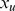 denotes the (1×*k*) vector whose values are functions of inputs for the i-th company in the t-th time period,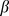 is (1×*k*) vector of unknown parameters to be estimated, and 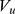
s are the error components of random disturbances, distributed i.i.d. *N (0*, 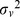 *)* and independent from 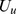. 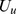
s are non-negative random variables associated with the technical inefficiency of production and can be expressed, following [22], as
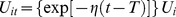
(2)where η is an unknown scalar parameter to be estimated, which determines whether inefficiencies are time-varying or time invariant, and *U_i_* s are assumed to be i.i.d. and truncated at zero of the *N (μ*, 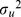 *)* distribution.

If *η* is positive, then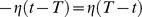 is positive for *t<T* and so, 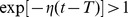, which implies that technical inefficiencies of companies decline over time. If *η* is zero, technical inefficiencies of industries remain constant; if it is negative, they increase over time.

The stochastic frontier model (1) was followed here to measure the technical efficiency of Dhaka Stock Market companies in Bangladesh. The maximum likelihood estimation (MLE) method was used to estimate the parameters of the stochastic frontier model. Using the composed error terms of the stochastic frontier model (1), the total variation in output from the frontier level of output, attributed to technical efficiency, is defined by 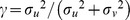. In truncated and half-normal distributions, the ratio of industry-specific variability to total variability, *γ*, is positive and significant, implying that company-specific technical efficiency is important for examining the total variability of output produced. This was done by calculating the maximum likelihood estimates for the parameters of the stochastic frontier model with the computer program FRONTIER Version 4.1 [23].

### Data Sources

The data collected from Dhaka Stock Exchange (DSE) market belongs to 94 companies in Bangladesh for the period of 2000–2008. The DSE market includes 22 categories of companies, of which the following 13 categories were covered: Banks, Investments, Engineering, Food & Allied Products, Fuel & Power, Textiles, Pharmaceuticals & Chemicals, Service & Real Estate, Cement, Tannery Industries, Ceramic Industry, Insurance and Miscellaneous. Of the companies studied, 58 belong to non financial sector and 36 to financial sector. In short, it can be said that the data represents the overall market.

### Variables Construction

Individual Return (Y): For this study, individual company's return was taken as a dependent variable. DSE prepares individual company's daily closing price by using which the return of individual company is calculated as follows:
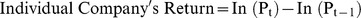
where P_t_ = closing price at period t, P_t-1_ = closing price at period t-1 and ln = natural log.

To obtain individual company's return, no adjustment was made of company's dividend, bonus and right issues, because many researchers confirmed that their conclusions remained unchanged regardless of making or not making such adjustment [24], [25]. Taking logarithmic returns is justified both theoretically and empirically. Theoretically, logarithmic returns are analytically more tractable when linking returns over longer intervals. Empirically, they are more likely to be normally distributed, which is a prior condition for standard statistical techniques [26].

Market Return(X_1_): DSE prepares daily price index from the daily weighted-average price of daily transaction of each stock and terms it as the “All Share Price Index”. Market return is calculated as follows:
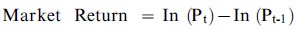
where P_t_ = price index at period t, P_t-1_ = price index at period t-1 and ln = natural log.

Market Capitalization(X_2_): Market Capitalization is the total value of a company's issued share capital as determined by its share price in the stock market. It is calculated as the number of ordinary shares in issue multiplied by the previous day's closing share price and is expressed in millions. The formula is as follows:
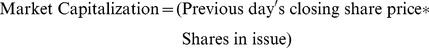
Book to Market Ratio(X_3_): The book value of a company is total assets minus intangible assets and liabilities. Here the company's net asset value per share was taken as the book value of that company. The market value is the share value in the current market price. After establishing the book value and the market value of a company, the book to market ratio was obtained by simply dividing the former by the latter:
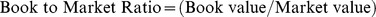
Market Value(X_4_): The total monetary value of securities traded in a specific period is called the market value of that period. The market value was calculated by multiplying share price with the number of securities traded, as shown below:
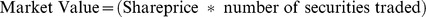



### Empirical Stochastic Frontier Model

The Cobb-Douglas stochastic frontier production with distributional assumption was selected to assess the technical efficiency of companies in DSE market, because panel data was used in this study and the sample number was not very high. Besides, in its generalized form, it is a simple tool that can be handled easily even for multiple inputs [27].The empirical version of stochastic frontier model (1) with the specification of Cobb-Douglas functional form can be expressed thus with the decomposed errors:
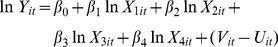
(3)where, the subscripts i and t represent the i-th company and the t-th year of observation, respectively, and *i* = 1,2,…,94 and *t* = 1,2,….,9; *Y_it_* represents the individual return, *X_1it_* the market return, *X_2it_* market capitalization, *X_3it_* book to market ratio and *X_4it_* market value.

“ln” refers to the natural logarithm; the *β_i_*'s are unknown parameters to be estimated; *V_it_* follows *N (0*, 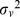 *)* and *U_it_* follows half-normal or truncated normal distribution at zero and guarantees inefficiency to be positive only.

The technical efficiency for the i-th company in the t-th year can be defined in the context of stochastic frontier model (1) as follows [28]:
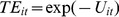
(4)
*U_it_* denotes the specifications of the inefficiency model in equation (2).

### Tests of Hypothesis

A series of formal hypothesis tests were conducted to determine the distribution of the random variables associated with the existence of technical inefficiency and the residual error term. If the null hypothesis involves *γ* = 0, it expresses that technical inefficiency effects are not present in the model. The half-normal distribution is a special case of the truncated normal distribution and implicitly involves the restriction *H_0_:μ = 0*. The hypothesis shows that efficiency, invariant over time (i.e. *η* = 0), will be tested. These are tested by imposing restrictions on the model and using the generalized likelihood-ratio statistic (λ) to determine the significance of the restriction. The generalized likelihood ration statistic is defined by
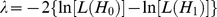
(5)where 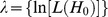 and 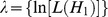 are the values of the log-likelihood function for the frontier model under the null and alternative hypotheses.

## Results

### Ordinary Least Square Estimation

The ordinary least square (OLS) estimates of the parameters of Cobb-Douglas production function were obtained first by grid search. These estimates were used to estimate the maximum likelihood estimates of the parameters of Cobb-Douglas stochastic frontier production model. The ordinary least square estimates show the average performance of the sample companies that were presented in Table 1. From the analysis, it is observed that the coefficients of market return, market capitalization, book to market ratio and market value were statistically significant in the stock market. The results indicate that these input variables significantly affect the individual company's return, listed in the DSE market. The parameter σ is positive, which indicates that the observed output differs from frontier output owing to factors which are within the controls of the stock market.

**Table 1:** OLS Estimates of the Cobb-Douglas Stochastic Frontier Production Function.

Variables	Parameters	Coefficients	S.E	t-value
Constant	*β_0_*	-0.4911@	0.3940	−1.247
Market Return	*β_1_*	0.4553^*^	0.0535	8.507
Market Capitalization	*β_2_*	−0.1548^*^	0.0374	−4.135
Book to Market Ratio	*β_3_*	−0.0596^*^	0.0139	−4.281
Market Value	*β_4_*	0.2305^*^	0.0335	6.884
Sigma-squared		0.0855		
Log likelihood function		−157.6330		

*, **, *** Significance level at 1%, 5% and 10% consecutively,

@means insignificant,

S.E = Standard Error.

### Estimation of Stochastic Frontier Model

The maximum-likelihood estimates for the parameters for the time-variant and time-invariant Cobb-Douglas stochastic frontier production function with the assumptions are presented in Tables 2 and 3 respectively. The results in Table 2 show that the estimates of the parameters with time-varying inefficiency effects for truncated and half-normal distributions are respectively 0.3873 and 0.4112 for market return input, −0.1651 and −0.1467 for market capitalization input, −0.0771 and −0.0750 for book to market ratio input, and 0.2925 and 0.2806 for market value input. The MLE of market return, market capitalization and book to market ratio in half-normal distribution are found to be higher than those in truncated normal distribution; only the MLE of market value in half-normal distribution is smaller than that in truncated normal distribution. Nonetheless, the estimated values of the parameters of the Cobb-Douglas frontier production function obtained with the two distributional assumptions are almost similar. The log likelihood functional values also are similar for the two distributions. For truncated normal distribution, γ was estimated to be 0.7632 and for half-normal distribution 0.4770; both the values are significant. It can be interpreted that 76 percent of random variation for truncated normal distribution, as also 47 percent for half-normal distribution, in stock market returns is due to inefficiency. This can also be interpreted that the 76 percent variation in output among the companies is due to the differences in technical efficiency for truncated normal distribution and the 47 percent variation to the differences in technical efficiency for half-normal distribution. It is evident from Table 2 that the estimates of σ are 0.3286 and 0.1522 for truncated and half-normal distribution respectively, which are significantly different from Zero indicating a good fit and correctness for the assumptions of truncated and half-normal distributions. The estimates for the parameters of the time varying inefficiency model in Table 2 indicate that because the estimates for η parameter are negative, the technical inefficiency effects tend to increase over time.

**Table 2:** Maximum-Likelihood Estimates of the Cobb-Douglas Stochastic Frontier Production Function with Time-variant.

			Truncated Normal			Half Normal	
Variables	Parameters	Coefficients	S.E	t-value	Coefficients	S.E	t-value
Constant	*β_0_*	−1.3776^*^	0.3830	−3.597	−1.6140^*^	0.4331	−3.727
Market Return	*β_1_*	0.3873^*^	0.0449	8.630	0.4112^*^	0.0533	7.719
Market Capitalization	*β_2_*	−0.1651^*^	0.0368	−4.486	−0.1467^*^	0.0402	−3.647
Book to Market Ratio	*β_3_*	−0.0771^*^	0.0145	−5.336	−0.0750^*^	0.0151	−4.980
Market Value	*β_4_*	0.2925^*^	0.0335	8.740	0.2806^*^	0.0393	7.145
Sigma-squared	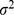	0.3286^*^	0.0722	4.548	0.1522^*^	0.0303	5.028
Gamma	γ	0.7632^*^	0.0605	12.6053	0.4770^*^	0.1067	4.473
Mu	μ	−1.0015^**^	0.4504	−2.224	0	0	0
Eta	η	−0.4964^*^	0.0919	−5.402	−0.4657^*^	0.1051	−4.432
Log-likelihood		−149.0902			−152.3256		

*, **, *** Significance level at 1%, 5% and 10% consecutively, @ means insignificant, S.E = Standard Error.

**Table 3:** Maximum-Likelihood Estimates of the Cobb-Douglas Stochastic Frontier Production Function with Time-invariant.

			Truncated Normal			Half Normal	
Variables	Parameters	Coefficients	S.E	t-value	Coefficients	S.E	t-value
Constant	*β_0_*	-0.4332@	0.3945	−1.098	-0.4359@	0.3923	−1.111
Market Return	*β_1_*	0.4559^*^	0.0531	8.587	0.4551^*^	0.0530	8.591
Market Capitalization	*β_2_*	−0.1555^*^	0.0373	−4.166	−0.1549^*^	0.0370	−4.183
Book to Market Ratio	*β_3_*	−0.0621^*^	0.0147	−4.230	−0.0617^*^	0.0147	−4.191
Market Value	*β_4_*	0.2308^*^	0.0333	6.932	0.2302^*^	0.0331	6.951
Sigma-squared	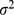	0.0990^*^	0.0260	3.811	0.0871^*^	0.0052	16.9038
Gamma	γ	0.1572@	0.2428	0.6475	0.0388@	0.0469	0.8280
Mu	μ	-0.2495@	0.5387	−0.4631	0	0	0
Eta	η	0	0	0	0	0	0
Log-likelihood function		−157.1236			−157.2972		
Mean efficiency		0.9557			0.9553		

*, **, *** Significance level at 1%, 5% and 10% consecutively,

@means insignificant,

S.E = Standard Error.

As regards Cobb-Douglas stochastic frontier production function with time-variant, the maximum-likelihood estimates of the coefficients of market return, market capitalization, book to market ratio and market value are found to be significant at 1% level for both distributions. These results indicate that the input variables significantly affect the amount of return in the individual companies listed in the DSE market for both truncated normal and half normal distributions. A significant negative relationship is observed between share returns and market capitalization, which supports some findings [29]–[31], but contradicts others [32], because subsequent studies show a positive relationship between market capitalization and share returns. Also, there is a significant negative relationship between book-to-market ratio and stock returns which contradicts the emerging market research finding that a significant positive relationship exists between book-to-market ratio and share returns. The market return shows significant relationship with the stock returns which means that if the overall market rises, then the return of individual companies will increase, and if the overall market falls, then the return of individual companies will decrease. The other input variable, namely the market value also shows significant relationship with the stock returns which means that if the market value of individual company shows upper trend, then the return of that company will increase, whereas if it shows lower trend, then the return of that company will decrease.

The results in Table 3 show that the maximum-likelihood estimates of the parameters with time-invarying inefficiency effects for truncated and half-normal distributions are respectively 0.4559 and 0.4551 for market return input, −0.1555 and −0.1549 for market capitalization input, −0.0621 and −0.0617 for book to market ratio input, and 0.2308 and 0.2302 for market value input. The MLE of market capitalization and book to market ratio in half-normal distribution is higher than that in truncated normal distribution; the MLE of market return and market value in half-normal distribution is smaller than that in truncated normal distribution. The log likelihood functional values of the two distributions are rather similar. In the case of both truncated and half-normal distributions, the values of γ are found to be positive and insignificant, whereas in the case of time variant truncated and half-normal distributions, they are found to be positive, yet significant, thus demonstrating that over time there could be technical inefficiency in the companies of DSE market in Bangladesh. The η parameter is restricted to zero in the model with time-invarying inefficiency effects.

In the case of Cobb-Douglas stochastic frontier production function with time-invariant, the maximum-likelihood estimates of the coefficients of market return, market capitalization, book to market ratio and market value are found to be significant at 1% level for both the distributions. These results also indicate that the input variables studied for time-invariant case significantly affect the amount of return in individual companies listed in the DSE market for both truncated normal and half normal distributions. The market return and market value show significant positive relationship with the stock returns. The ratio of the other two input variables, namely market capitalization and book-to-market, ratio shows significant negative relationship with the stock returns. These findings are similar to those of time-variant case in terms of the relationship between input variables and stock returns.

### Year-wise Mean Efficiency of Companies: Results from Truncated Normal and Half-Normal

**Figure 1:**
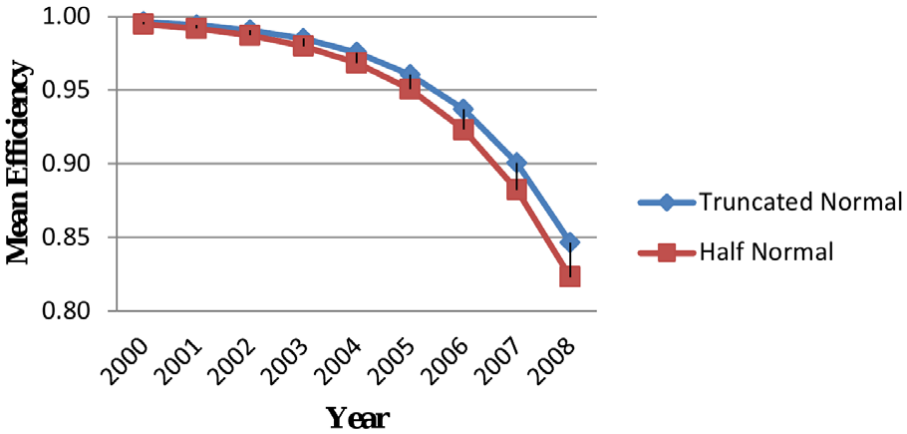
Year-wise mean efficiency by distribution.

The year-wise average efficiency of 94 companies in DSE market in terms of distributions with time-variant is presented in Table 4 and Figure 1. From these figures, it is observed that the mean efficiency values are in the range of 0.8467 to 0.9966 for truncated normal distribution and 0.8232 to 0.9950 for half-normal distribution. The mean technical efficiency of the companies during the period 2000–2008 is 0.9542 for truncated normal distribution and 0.9448 for half normal distribution. This implies that 95 percent and 94 percent of potential outputs were being realized by the companies of DSE market according to truncated normal distribution and half-normal distribution respectively. The truncated normal distribution gave higher technical efficiency estimates than did the half normal distribution.

**Table 4:** Year-wise Average Efficiency of Companies in Dhaka Stock Exchange by distribution with Time-variant.

Year	Truncated Normal	Half Normal
2000	0.9966	0.9950
2001	0.9944	0.9921
2002	0.9909	0.9874
2003	0.9851	0.9801
2004	0.9757	0.9686
2005	0.9607	0.9507
2006	0.9371	0.9234
2007	0.9007	0.8824
2008	0.8467	0.8232
**Mean**	0.9542	0.9448

The technical efficiency decreased in both distributions over the period 2000–2008. It is argued by [33] that a combination of factors like insufficient financial information, thin and discontinuous trading, trust on price momentum and manipulation by the market makers create the conditions that lead to the decreasing trend of efficiency. The reason of declining trend of efficiency is also the poor institutional infrastructure, weak regulatory framework, lack of supervision, poor corporate governance, slow development of the market infrastructure and lack of transparency of market transactions [17]. A lower trend of efficiency on emerging markets might be due to common characteristics of loose disclosure requirements, thinness and discontinuity in trading [34] or due to the institutional factors such as market fragmentation, trading and reporting delays and absence of official market makers [35] or due to the delay in operations and high transaction cost, thinness of trading in the market [36].

### Group-wise Technical Efficiency: Results from Truncated Normal and Half-normal with Time-variant

**Figure 2:**
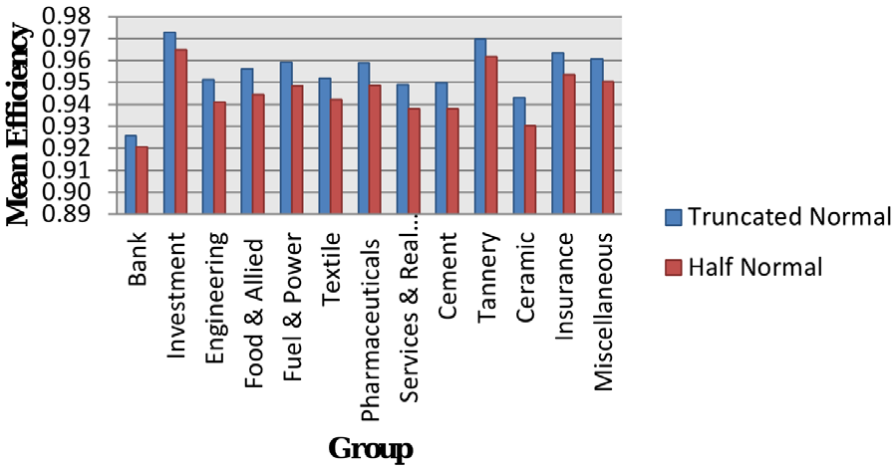
Group-wise mean efficiency by distribution with time-variant.

Group-wise technical efficiency of both truncated normal and half-normal models with time-variant is shown in Table 5 and Figure 2. The technical efficiency varies among different groups of DSE market: For truncated normal distribution, it ranges from a minimum of 0.9259 for Bank-group to a maximum of 0.9727 for Investment-group; for half-normal distribution, it ranges from a minimum of 0.9207 for Bank-group to a maximum of 0.9649 for Investment-group. The actual range is 0.0468 for truncated normal distribution and 0.0442 for half-normal distribution.

**Table 5:** Group-wise Mean Efficiency in Dhaka Stock Exchange by distribution with Time-variant.

Group	Truncated Normal	Half Normal
Bank	0.9259	0.9207
Investment	0.9727	0.9649
Engineering	0.9512	0.9409
Food & Allied	0.9560	0.9445
Fuel & Power	0.9591	0.9486
Textile	0.9519	0.9421
Pharmaceuticals	0.9587	0.9486
Services & Real Estate	0.9489	0.9378
Cement	0.9499	0.9379
Tannery	0.9697	0.9616
Ceramic	0.9429	0.9304
Insurance	0.9635	0.9533
Miscellaneous	0.9605	0.9504

Based on these results, it is concluded that the value of technical efficiency is high for Investment-group and low for Bank-group, in comparison to other groups in DSE market. It is further observed that technical efficiencies for different groups are greater in case of truncated normal distribution than those of half-normal distribution.

### Group-wise Technical Efficiency: Results from Truncated Normal and Half-normal Models with Time-invariant

**Figure 3:**
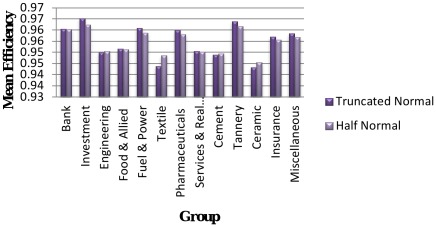
Group-wise mean efficiency by distribution with time-invariant.

Results in respect of group-wise technical efficiency of both truncated normal and half-normal models with time-invariant are presented in Table 6 and Figure 3. From these results it can be seen that technical efficiencies vary among different groups of DSE market: in the case of truncated normal distribution, it ranges between a low of 0.9433 for Ceramic-group and a high of 0.9653 for Investment-group; in the case of half-normal distribution, it ranges between a low of 0.9455 and a high of 0.9625 for the same groups. The actual range is 0.0220 for truncated normal distribution and 0.0170 for half-normal distribution. The Bank-group technical efficiency is found to be the same (Efficiency = 0.9605) for both truncated normal and half-normal distributions.

**Table 6:** Group-wise Mean Efficiency in Dhaka Stock Exchange by distribution with Time- invariant.

Group	Truncated Normal	Half Normal
Bank	0.9605	0.9605
Investment	0.9653	0.9625
Engineering	0.9501	0.9506
Food & Allied	0.9516	0.9514
Fuel & Power	0.9610	0.9589
Textile	0.9439	0.9486
Pharmaceuticals	0.9601	0.9582
Services & Real Estate	0.9506	0.9504
Cement	0.9490	0.9495
Tannery	0.9640	0.9618
Ceramic	0.9433	0.9455
Insurance	0.9572	0.9557
Miscellaneous	0.9585	0.9569

From Figure 3, it is concluded that the values of technical efficiency for engineering, textile, cement and ceramic groups are higher in half-normal distribution than those in truncated normal distribution. This result is at variance with the result of group-wise technical efficiency with time-variant. In time-invariant situation, it is observed that technical efficiencies of eight groups (Investment, Food & Allied, Fuel & Power, Pharmaceuticals, Services & Real Estate, Tannery, Insurance and Miscellaneous) are greater in truncated normal distribution than those in half-normal distribution.

### Results from Hypothesis Test

Formal tests of various hypotheses were carried out using the Likelihood Ratio (L-R) statistics (5) presented in Table 7. The first null hypothesis, *H_0_: γ = 0* specifies that there are no technical inefficiency effects in the model. Having rejected the hypothesis, it is concluded that there are technical inefficiency effects in the model. This implies that the technical inefficiency effects associated with the companies of Bangladesh Stock Market are significant. The technical inefficiency effects, having half-normal distribution, were tested by the null hypothesis *H_0_: μ = 0*. In this study, this hypothesis, which indicates that the truncated (at zero) normal distribution is preferable to half normal distribution for technical inefficiency effect, was rejected. The hypothesis *H_0_: η = 0*, which indicates that the technical inefficiency effect varied significantly over time, was also rejected.

**Table 7:** Generalized Likelihood-Ratio Test of Hypothesis of the Stochastic Frontier Production Model.

Null hypothesis	Log-likelihood function	Test Statistic	Critical value^*^	Decision
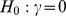	−157.6330	17.0856	2.706	Reject
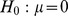	−152.3256	6.4708	2.706	Reject
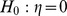	−157.1236	16.0668	2.706	Reject

Notes: All critical values are at 5% level of significance.

The list of companies is shown in Table 8.

**Table 8:** List of Companies considered in this study.

Serial No.	Company's Name	Group	Serial No.	Company's Name	Group
1	AB Bank	Bank	48	Apex Spinning	Textile
2	City Bank	Bank	49	Delta Spinners	Textile
3	IFIC Bank	Bank	50	Sonargaon Textiles	Textile
4	Islami Bank	Bank	51	Prime Textile	Textile
5	NBL	Bank	52	H.R.Textile	Textile
6	Uttara Bank	Bank	53	Ambee Pharma	Pharmaceuticals
7	Eastern Bank	Bank	54	Beximco Pharma	Pharmaceuticals
8	Al-Arafah IB	Bank	55	Glaxo SmithKline	Pharmaceuticals
9	ICB	Bank	56	ACI Limited	Pharmaceuticals
10	IDLC	Bank	57	Renata Ltd	Pharmaceuticals
11	United Leasing	Bank	58	Reckitt Benckiser	Pharmaceuticals
12	Uttara Finance	Bank	59	The Ibn Sina	Pharmaceuticals
13	1stICB M.F.	Investment	60	Beximco Synthetics	Pharmaceuticals
14	2nd ICB M.F	Investment	61	Libra Infusions	Pharmaceuticals
15	3rd ICB M.F.	Investment	62	Square Pharma	Pharmaceuticals
16	4th ICB M.F.	Investment	63	Imam Button	Pharmaceuticals
17	5th ICB M.F.	Investment	64	Samorita Hospital	Services & Real Estate
18	6th ICB M.F.	Investment	65	Eastern Housing	Services & Real Estate
19	7th ICB M.F.	Investment	66	Heidelberg Cement	Cement
20	8th ICB M.F.	Investment	67	Confidence Cement	Cement
21	1st BSRS	Investment	68	Meghna Cement	Cement
22	Aftab Automobiles	Engineering	69	Aramit Cement	Cement
23	Olympic Industries	Engineering	70	Apex Tannery	Tannery
24	Bangladesh Lamps	Engineering	71	Bata Shoe	Tannery
25	Eastern Cables	Engineering	72	**A**pex Adelchy Ft.	Tannery
26	Monno Jutex	Engineering	73	Monno Ceramic	Ceramic
27	Monno Stafllers	Engineering	74	Standard Ceramic	Ceramic
28	Singer Bangladesh	Engineering	75	BGIC	Insurance
29	Atlas Bangladesh	Engineering	76	Green D.Ins.	Insurance
30	BD.Autocars	Engineering	77	United Ins.	Insurance
31	Quasem Drycells	Engineering	78	Peoples Ins.	Insurance
32	National Tubes	Engineering	79	Eastern Ins.	Insurance
33	Bd.Thai Aluminium	Engineering	80	Janata Ins	Insurance
34	Anwar Galvanizing	Engineering	81	Phoenix Ins	Insurance
35	Kay & Que	Engineering	82	Eastland Ins	Insurance
36	National Polymer	Engineering	83	Central Ins	Insurance
37	Apex Foods	Food & Allied	84	Karnaphuli Ins	Insurance
38	Bangas	Food & Allied	85	Rupali Ins	Insurance
39	BATBC	Food & Allied	86	Federal Ins	Insurance
40	National Tea	Food & Allied	87	Reliance Ins	Insurance
41	AMCL (Pran)	Food & Allied	88	Purabi G.Ins	Insurance
42	Rahima Food	Food & Allied	89	Pragati Ins.	Insurance
43	BOC	Fuel & Power	90	Aramit	Miscellaneous
44	Padma Oil Co.	Fuel & Power	91	GQ Ball Pen	Miscellaneous
45	Saiham Textile	Textile	92	Usmania Glass	Miscellaneous
46	Desh Garmants	Textile	93	Savar Ref.	Miscellaneous
47	Bextex Limited	Textile	94	BEXIMCO	Miscellaneous

## Discussion

The study identifies the general determinants of share returns in Dhaka Stock Exchange (DSE). Because of the similar types of characteristics such as thin trading, volatility, small number of securities listed, investors' attitude towards investment strategy, Dhaka stock market seems to be like some other emerging markets such as the Indian market, the Johannesburg Stock Exchange, the Kuwaiti stock market, some of the Middle Eastern markets.

The results suggest that the input variables, such as market return, market capitalization, book-to-market ratio and market value have significant influence on share returns. This indicates that all the input variables are important for companies in DSE market. It is observed through several tests that technical inefficiency effects are significant which implies that their association with the companies of Bangladesh Stock Market is significant. For technical inefficiency effect, truncated normal distribution is found to be preferable to half normal distribution. It is found that the technical efficiency rate in Bangladesh stock market decreased gradually over time. For this study, group-wise technical efficiency of Dhaka Stock Exchange was also analyzed. In the time-variant situation of group-wise technical efficiency, the investment group gives the highest technical efficiency and the bank group the lowest technical efficiency for both truncated normal and half-normal distributions. For a similar analysis in time-invariant situation, the investment group gives the highest technical efficiency and the ceramic group the lowest technical efficiency for both truncated normal and half-normal distributions.

The results of this study are of great interest to academics, policy makers, and local and foreign companies, both listed and unlisted. Also, they have important practical implications to different capital market participants such as investors, managers and regulatory authorities. As the presence of the decreasing technical efficiency of the DSE market, it informs the regulators and policy makers that appropriate measures should be taken to increase the technical efficiency in the market.

Moreover, globalization of world economy has created an enormous opportunity for the investors to diversify their portfolios across the globe. As a result, examining the efficiency and characteristics of DSE markets would be of great benefit to investors at home and abroad. Finally, it may also be useful to international organizations (such as the World Bank, IMF, WTO) and governments of partners who are interested in the development of capital markets in third-world countries. The stock market can thus play an important role in inducing economic growth in Bangladesh by channeling investments from the public.

## References

[1] Berger AN, Humprey DB (1997). Efficiency of Financial Institutions: International Survey and Directions for Future Research.. European Journal of Operational Research.

[2] Ferrier GD, Lovell CAK (1990). Measuring cost efficiency in banking: Econometric and linear programming evidence.. Journal of Econometrics.

[3] Pastor M, Perez F, Queseda J (1997). Efficiency analysis in banking firms: An international comparison.. European Journal of Operational Research.

[4] Resti A (1997). Evaluating the cost-efficiency of the Italian banking system: What can be learned from the joint application of parametric and non-parametric techniques.. Journal of Banking and Finance.

[5] Bauer P, Berger AN, Ferrier G, Humphrey DB (1998). Consistency conditions for Regulatory analysis of financial institutions: A comparison of frontier efficiency methods.. Journal of Economics and Business.

[6] Altunbas Y, Evans L, Molyneux P (2001). Bank ownership and efficiency.. Journal of Money, Credit, and Banking.

[7] Maudos J, Pastor M, Perez F, Queseda J (2002). Cost and profit efficiency in European banks.. J Journal of International Financial Markets, Institutions and Money.

[8] Weill L (2004). Measuring cost efficiency in European banking: A comparison of frontier techniques.. Journal of Productivity Analysis.

[9] Fecher F, Kesler D, Perelman S, Pestieau P (1993). Productive performance of the French insurance industry.. Journal of Productivity Analysis.

[10] Cummins JD, Zi H (1998). Comparison of frontier efficiency methods: An application to the U.S. life insurance industry.. Journal of Productivity Analysis.

[11] Aigner D, Lovell CAK, Schmidt P (1977). Formulation and Estimation of Stochastic Frontier Production Function Models.. Journal of Econometrics.

[12] Kirkley JE, Squires D, Strand IE (1995). Assessing Technical Efficiency in Commercial Fisheries: The Mid-Atlantic Sea Scallop Industry.. American Journal of Agricultural Economics.

[13] Greene, Fried HO, Lovell CAK, Schmidt SS (1993). The Econometric Approach to Efficiency Analysis.. The Measurement of Productive Efficiency: Techniques and Applications.

[14] Yuengert A (1993). The Measurement of Efficiency in Life Insurance: Estimates of a Mixed Normal-Gamma Error Model.. Journal of Productivity Analysis.

[15] Mester LJ (1996). A Study of Bank Efficiency Taking into Account Risk preferences.. Journal of Banking and Finance.

[16] Hjalmarsson L, Kumbhakar S, Heshmati A (1996). DEA, DFA and SFA: A Comparison.. Journal of Productivity Analysis.

[17] Uddin MGS, Khoda AKMN (2009). An Empirical Examination of Random Walk Hypothesis for Dhaka Stock Exchange: Evidence from Pharmaceutical Sector of Bangladesh.. International Research Journal of Finance and Economics.

[18] Alam MI, Hasan T, Kadapakkam P (1999). An Application of Variance Ratio Test to Five Asian Stock Markets.. Review of Pacific Basin Financial Markets and Policies.

[19] Hassan MK, Maroney NC (2004). Thin trading, non-linearity and market efficiency of a small emerging stock market: Evidence from Bangladesh.. International Journal of Applied Business and Economic Research.

[20] Uddin MGS, Alam MM (2007). The Impacts of Interest Rate on Stock Market: Empirical Evidence from Dhaka Stock Exchange.. South Asian Journal of Management Science.

[21] Kasman S, Turgutlu E (2007).

[22] Battese GE (1992). Frontier Production Function and Technical Efficiency: A Survey of Empirical Applications in Agricultural Economics.. Agricultural Economics.

[23] Coelli TJ (1996).

[24] Lakonishok J, Smidt S (1988). Are Seasonal Anomalies Real?. A Ninety Year Perspective Review Financial Studies.

[25] Fishe R, Gosnell T, Lasser D (1993). Good news, bad news, volume and the Monday effect.. Journal of Business Finance and Accounting.

[26] Strong N (1992). Modeling Abnormal Returns: A Review Article.. Journal of Business Finance and Accounting.

[27] Murthy KVB (2002). Arguing a Case for Cobb-Douglas Production Function.. Review of Commerce Studies.

[28] Battese GE, Coelli TJ (1988). Prediction of Grm-level technical efficiencies: With a generalized frontier production function and panel data.. Journal of Econometrics.

[29] Banz RW (1981). The relationship between return and market value of common stocks.. Journal of Financial Economics.

[30] Chan L, Hamao CY, Lakonishok J (1991). Fundamentals and stock returns in Japan.. Journal of Finance.

[31] Fama EF, French KR (1992). Cross-section of expected stock returns.. Journal of Finance.

[32] Claessens S, Dasgupta S, Glen J (1996). The cross-section of stock returns: Evidence from the emerging markets..

[33] Islam MM, Gomes JL (1999). The day-of-the-week effects in less-developed countries' markets: The case of Bangladesh.. International Advances in Economic Research.

[34] Errunza VR, Losq E (1985). The behavior of stock prices on LDC markets.. Journal of Banking and Finance.

[35] Butler KC, Malaikah SJ (1992). Efficiency and inefficiency in thinly traded stock markets: Kuwait and Saudi Arabia.. Journal of Banking and Finance.

[36] Nourredine K (1998). Behavior of stock prices in the Saudi Arabian Financial Market: Empirical research findings.. Journal of Financial Management & Analysis.

